# Life before limb: What about life after limb loss? A perspective from a low middle class income country: A Surgical Perspective

**DOI:** 10.12669/pjms.39.4.7038

**Published:** 2023

**Authors:** Nadeem Ahmed Siddiqui, Ammar Pirzada, Safna Naozer Virji

**Affiliations:** 1 Nadeem Ahmed Siddiqui, Department of Surgery, Aga Khan University, Karachi, Pakistan; 2 Ammar Pirzada, Department of Surgery, Aga Khan University, Karachi, Pakistan; 3 Safna Naozer Virji, Department of Surgery, Aga Khan University, Karachi, Pakistan

Amputations particularly in emergency settings are considered an important service delivery component of the bellwether procedures, which form the core of the global surgery initiative. The ongoing debate of making these procedures widely available at secondary care hospitals have caught attention from many proponents and advocates of health equity. But the question remains whether provision of service is the only criteria to gauge our success in terms of providing benefit to the patients?

Lower limb amputations are associated with significant disability, as there is considerable impairment of muscle strength, balance and walking ability after lower limb amputations. Furthermore, lower limb amputations have a psychosocial impact, with patients experiencing social embarrassment and ultimately social isolation. Also, due to productivity loss, these patients may suffer economic challenges due to the inability or limited ability to work. Keeping this in mind, serious introspection is needed particularly from a surgeon’s point of view. Lower limb amputation may save the life of the patient at a given point but it comes with a cost; a cost which is physical, financial, social and emotional or a combination of all of these.

Major lower limb amputations, worldwide range from 5.6 to 68.4 per 100,000^1^ and in a low middle income country (LMIC) like Pakistan, the major cause of limb amputations is due to complications of diabetes mellitus, followed by trauma.^2^ A systematic review revealed, that in Pakistan, the prevalence of diabetes is 14.62% and rising.^3^ Furthermore, in our country, poor compliance to medication among diabetics has resulted in a significantly greater incidence of diabetic foot ulcers which predisposes them to lower limb amputations in the long run.^4^ Another study conducted in 25 primary care centers in Pakistan determined a prevalence of 13.9% of patients with Type-2 diabetes being diagnosed with diabetic foot syndrome, and the rate of lower extremity amputation following diabetic foot syndrome has been estimated at 8-21%.^5^

In a country like ours, where health insurance is now finally being considered an integral part of job security and social well-being for permanent employees, a large chunk of our population still works as daily wagers who do not have this facility.^6^ Even insurances that cover these surgeries are only covering the operative cost, and this too may vary largely in an LMIC setting where hospital financial models are significantly variable ranging from completely subsidized government run facilities, to NGO managed hospitals, and lastly private hospitals.^7^ If this disparity was not enough, the area that gets neglected, not just by the insurance companies but also by the treating physicians is what happens to these patients once they are out of the hospital.

A general consensus is that, amputations in a non-welfare state like ours, leaves the patient and his/her family in a precarious space where patient care, return to normalcy and attaining financial independence becomes extremely difficult. This may sound a sweeping statement, but the surgeon’s fraternity who operate on these patients and then see them on routine follow-up know this for a fact, that these patients and their family usually have a tough time in regaining both functional and financial independence.

A local study conducted in 2008 determined the mean cost of trans-tibial and trans-femoral amputations to be PKR 46,182.^2^ This study is over a decade old and it does not take into consideration the cost of rehabilitation and prosthesis. A more recent retrospective review conducted in 2019, of 63 patients from Faisalabad concluded that the operative cost alone of major amputations in a period of six months was USD 27,568 in a government run hospital that amounts to USD 437 per patient, which is a significant number.^8^ Again this did not take into account the postoperative period, highlighting the lack of assessment of the financial impact for diabetic foot syndrome in Pakistan and its functional and financial outcomes. We believe that the cost estimates done for these patients underestimates the true financial impact which the patient has to bear in real life. Even after getting the procedure done, the patient needs some assistance like post-operative dressings, physiotherapy and pain medicines which are not included in cost estimations. Similarly many patients have to sell their property, household items or jewellery to be able to meet the costs during the treatment. This issue gets more complicated if the patient is the sole breadwinner for the family and loses his/her job or has to take long leaves without pay during the course of treatment. Similarly the magnitude of social and emotional impact which the patient and family members have to bear is enormous. Limited functional status and dependency on family members for routine daily activities brings significant emotional burden. Effect of lower limb amputations on intimate relationships have not been studied in our part of the world.

So how does one go about it? Should amputation be avoided at all costs? The answer to this lies in the famous quote “LIFE BEFORE LIMB”. Can amputation be preempted? This possibly can be addressed. But before coining the solution to any problem, there first has to be acknowledgement of the problem.

Unfortunately, till date we do not have a validated tool to assess the functional and financial outcomes of major lower limb amputations in our population. Yes, there are international studies, mostly from the developed world, that have developed and validated tools like Amputee Single Item Mobility Measure (AMPSIMM) and AMPREDICT-Mobility tool, to determine the functional outcomes of patients after major lower limb amputations, but these models cannot simply be replicated here, prominently, because of the financial and healthcare inequity that we have in our country.^9^,^10^ Affording patients may not face much difficulty but individuals belonging to the poor socioeconomic class look up to NGOs and other donors to help them navigate through this difficult phase. Financial models, rehabilitation facilities and functional expectations are different in our part of the world.

All these factors make us think, that for our region we need to have thorough objective evidence of a true estimation of social, physical and financial impact after amputation by using a validated tool that is indigenous and answers our queries. Once we have the true understanding of the problem, only then interventions can be planned and funded by the higher authorities to preempt and avoid these major life changing procedures. We hope that this letter serves as food for thought for the surgeons and physicians dealing with such patients and they start thinking beyond, about the life of an amputee with above mentioned post amputation physical, social and financial concerns.
